# Map-Like Representations of an Abstract Conceptual Space in the Human Brain

**DOI:** 10.3389/fnhum.2021.620056

**Published:** 2021-02-02

**Authors:** Levan Bokeria, Richard N. Henson, Robert M. Mok

**Affiliations:** MRC Cognition and Brain Sciences Unit, University of Cambridge, Cambridge, United Kingdom

**Keywords:** conceptual space, concept learning, semantics, categorization, navigation, grid cells, entorhinal cortex

## Introduction

Much of higher cognition involves abstracting away from sensory details and thinking conceptually. How do our brains learn and represent such abstract concepts? Recent work has proposed that neural representations in the medial temporal lobe (MTL), which are involved in spatial navigation, might also support learning of higher-level knowledge structures (Behrens et al., 2018; Bellmund et al., 2018). Under this view, a range of MTL neurons such as place cells, grid cells, and head-direction cells may support the ability to mentally “navigate” through conceptual spaces. This extends the original proposal by Tolman (1948) that people construct “cognitive maps” that support broad psychological functions, and offers the exciting potential of understanding the cognitive processes that underlie category learning, reinforcement learning, and spatial navigation under a single unified framework.

These ideas are supported by findings that neural representations in the MTL, as well as the medial prefrontal cortex (mPFC), are involved in “navigation” of simple two-dimensional spaces of visual stimuli (Constantinescu et al., 2016; Theves et al., 2019, 2020), social spaces (Tavares et al., 2015; Park et al., 2020), and odor spaces (Bao et al., 2019). A recent study in the Journal of Neuroscience (Viganò and Piazza, 2020) takes this research further by suggesting that the entorhinal cortex (EHC) and the mPFC are capable of mapping not only sensory spaces, but also abstract semantic spaces. In this opinion piece, we first describe the paradigm and results of Viganò and Piazza (2020), as well as the importance of their findings for the field. We then raise several methodological concerns and suggest changes to the paradigm to address these issues. Finally, we discuss potential future research directions including experimental and modeling approaches to tackle outstanding questions in the field.

## Experimental Paradigm and Results

**Figure 1:**
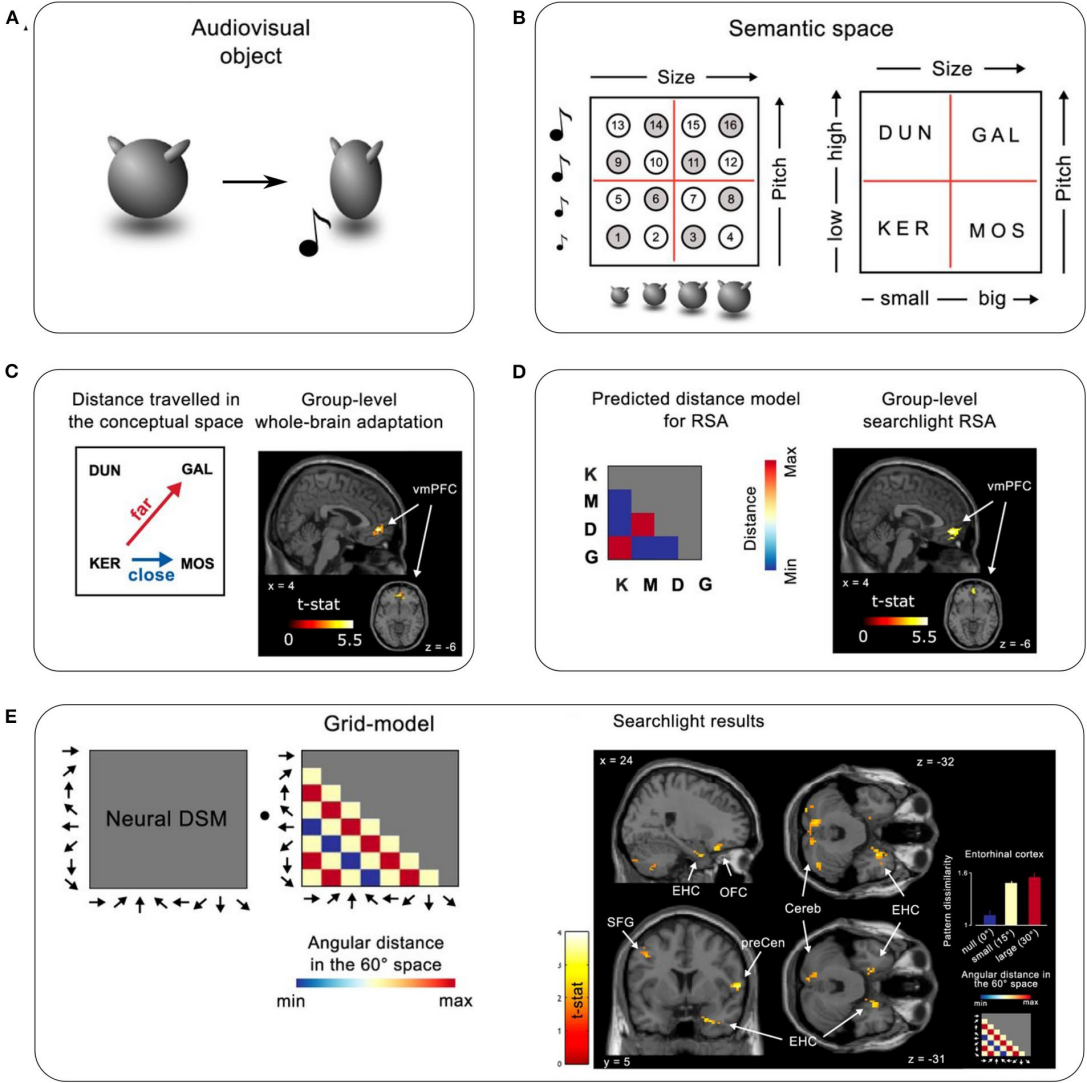
Task and results summary. **(A)** An example audiovisual object used in the experiment. Each object was of a particular size and made a sound of a specific pitch as it visually compressed on the screen. **(B)** Left: Illustration of the 16 objects in a 2D audiovisual sensory space defined by size and pitch. Right: Objects were assigned one of four category labels (meaningless pseudowords), which defined the abstract semantic/category space on top of the sensory space. Objects marked with white and gray circles were both used in the behavioral sessions; only the ones marked in gray were used in the fMRI sessions. **(C,D)** Brain representations coding semantic distance. **(C)** Left: Illustration of greater distances in semantic space between diagonal categories (here KER—GAL) than non-diagonal distances (here KER—MOS). Right: Whole-brain fMRI-adaptation analysis performed on post-learning data revealed a significant cluster in the mPFC. **(D)** Left: Representational similarity analysis (RSA) using a model based on distances between categories. Right: Whole-brain RSA searchlight revealed a significant cluster in the mPFC. **(E)** Grid-like representations of semantic space. Left: Assuming a grid-like representation in semantic space, a specific pattern of activation across different movement directions should be expected (where movement refers to transitions between stimuli on successive trials). A grid-model dissimilarity matrix represents the predicted pairwise dissimilarities between voxel activity patterns evoked by the different directions as a function of their angular distance in 60° periodic space. This grid-model is then correlated with the voxel activity patterns evoked by the different movement directions to reveal the presence of a grid-like response. Right: whole-brain searchlight results using the model-based grid-RSA. Maps are thresholded at *p* < 0.05 uncorrected for visualization. Bar plot inset shows the average dissimilarity between activity patterns evoked by different directions in the entorhinal cortex, varying as a function of angular distance modulo 60. The figure is adapted from Viganò and Piazza (2020), *Journal of Neuroscience*.

Viganò and Piazza (2020) employed a two-dimensional, multisensory space, in which each exemplar was characterized by the size of a visual image and the pitch of a concurrent auditory tone (Figures 1A,B). To assess how the brain organizes category knowledge in this space, they used functional magnetic resonance imaging (fMRI) to measure the distances and directions between the neural representations of exemplars, both before and after category learning. The experiment involved three phases: (1) a pre-learning fMRI task, (2) nine days of behavioral training on the category structure, and (3) a post-learning fMRI task. During pre-learning, participants performed a 1-back stimulus identity task on the exemplars and on verbal labels (the pseudowords KER, MOS, DUN, and GAL) that denoted the four later-to-be-learned categories (Figure 1B). During behavioral training, participants learned to map each exemplar to one of the four categories. The post-learning task was similar to the pre-learning task, except participants performed a 1-back category identity task, responding whenever an exemplar was followed by its corresponding category label or vice versa.

When searching for neural representations that related to the distances between categories, the authors utilized the fact that diagonal category pairs (such as DUN-MOS) differed in both dimensions and so were further apart than horizontal/vertical categories (such as KER-MOS) that differed in only one dimension (Figure 1C, left). They used fMRI-adaptation to detect sensitivity to small vs. large distances, and representational similarity analysis (RSA) to assess similarity between neural representations across categories (Figure 1D, left). Both analyses revealed a significant cluster in the mPFC that reflected greater distance between diagonal categories than horizontal/vertical categories post-learning, but not pre-learning (Figures 1C,D). An additional fMRI-adaptation analysis focusing on the EHC showed that the EHC also tracked category distances. Importantly, a control RSA based on distances between individual exemplars suggested that the mPFC was not sensitive to sensory distances between stimuli either pre- or post-learning, demonstrating its specificity to the more abstract category space.

The authors then used model-based RSA to test for direction-based grid-like neural codes for “movement” across the category space. A model was constructed to reflect the predicted similarities between all eight possible movement directions in the category space (“moving” from one trial to the next in the 1-back task). The similarities across directions were calculated assuming a 6-fold periodicity that would result from using a grid-like code (**Figure 1E**, left; see Bellmund et al., 2016; though note that only eight directions were sampled in this study). A whole-brain searchlight revealed a cluster in the right EHC consistent with this model (Figure 1E, right). This EHC signal was not sensitive to 4, 5, and 7-fold periodicities, and was only present post-learning. Notably, the authors did not find a direction-modulated signal in the mPFC.

Together, these results suggest that the neural mechanisms that underlie spatial processing are recycled to represent not just sensory spaces but also more abstract category spaces. Specifically, the mPFC and the EHC represent category distances, and the EHC tracks the movement directions in that space, possibly using the same grid-like codes found in spatial navigation tasks.

## Discussion

Despite its innovative paradigm, aspects of the experimental design warrant further consideration. Specifically, the pre- vs. post-learning tasks differed in nature (1-back stimulus identity vs. 1-back category identity), which could have introduced confounds such as differences in task difficulty—i.e., greater brain activity due to higher cognitive demand in the category task. This is one reason why the authors did not report a direct pre- vs. post-learning whole-brain comparison, even though such a contrast revealed the same mPFC cluster as reported in the paper (personal communication, April 9, 2020). One way to address this issue would be to run the 1-back stimulus identity task post- as well as pre-learning, attributing any changes in brain activity to the newly acquired category knowledge. This would allow a direct whole-brain comparison between pre- and post-learning stages, providing a better and more comprehensive test compared to the pre- post-learning comparison within the mPFC ROI identified by the analysis after learning (Figures 1C,D, right; also see Viganò and Piazza, 2020, Figures 2C,F). Indeed, the authors did run a post-learning 1-back stimulus identity task, but with fewer number of runs than pre-learning, undermining the power of the pre-post contrast (personal communication, April 9, 2020). Additionally, a post-learning 1-back stimulus identity task might not have activated the learned category knowledge since it is not required to perform the task. Thus, an alternative approach would be to introduce a within-participant “no-learning” control condition, where participants perform a pre-learning 1-back stimulus identity task and a post-learning 1-back category identity task on a second, distinct set of stimuli and category labels. Crucially, participants would not learn category-to-exemplar mappings in between the tasks (such that in the post-learning task, participants would need to guess the category to which exemplars belong). Then, one could examine distance and direction sensitivity that was unique to the “learned” space.

Another issue concerns the possibility that the direction-based, grid-like representations of concept space may be partially driven by more concept-level overlap in one direction than others. With four categories arranged in a square format, only a subset of directions across concepts could be sampled (0–315° in 45° steps), thereby omitting several key directions (60° and 120°) when testing for a 6-fold periodic signal (Figure 1E, left). Therefore, the significant correlation with the grid-model could have been mainly driven by the higher pattern similarity (lower dissimilarity) in the 180° direction. The issue is that the conditions that are compared in the 180° direction contained stimuli that shared two concept labels (e.g., DUN→GAL then GAL→DUN) whereas the other direction conditions only shared one concept label (e.g., one 45° movement is from DUN→KER then KER→GAL, sharing KER). If the brain region carries information about the concept, the 180° direction would show higher pattern similarity than the other directions whether or not there is an underlying grid-like signal. One hint of evidence supporting a grid-like representation the right EHC is that the pattern dissimilarities appear to increase from “zero” to “small” to “large” changes in the predicted grid-model RDM (Figure 1E, right, bar-plot inset). Although the numerical trend matches the 6-fold prediction, it is unclear whether the pattern dissimilarities for “small” and “large” are statistically distinguishable, and the main result could still be driven by the small dissimilarities in the “zero” condition (i.e., 180°). Future work could use a larger set of exemplars and concept labels to test a wider range of movement directions and hence provide further evidence for the emergence of a grid-like representation in concept space after learning.

It is notable that Viganò and Piazza (2020) did not find distance-based representations in the hippocampus, for either category or sensory spaces. Structures in the MTL have been implicated in a wide range of cognitive functions from spatial navigation to memory to concept formation. We provide a brief overview of these functions and speculate on the possible reasons for the absence of hippocampal involvement in the current study. First, the hippocampus is a region strongly implicated in spatial navigation, where place cells are thought to track an agent's physical location (O'Keefe and Nadel, 1978). Relatedly, human neuroimaging studies have implicated the hippocampus in tracking distances in virtual spaces (Deuker et al., 2016), conceptual spaces (Tavares et al., 2015; Theves et al., 2019, 2020) and graph-like relational knowledge structures (Garvert et al., 2017). The MTL has long been acknowledged for its role in learning and memory (Nadel and Moscovitch, 1997). MTL structures such as the hippocampus, EHC, parahippocampal cortex, and perirhinal cortex are involved in the integration of multiple sources of information such as object identity and spatio-temporal context into a coherent episodic event representation that can be consolidated into memory (Eichenbaum and Cohen, 2008; Knierim et al., 2014). Interestingly, the role of the MTL in semantic memory has been debated (Squire and Zola-Morgan, 1991; Mishkin et al., 1998; Duff et al., 2020) with some researchers arguing for the anterior temporal lobe (ATL) as the crucial region for representing semantic knowledge, supported by semantic deficits in semantic dementia (Ralph et al., 2016). Recent work also showed the involvement of MTL structures such as the hippocampus in concept learning and categorization (Davis et al., 2012; Mack et al., 2016; Bowman and Zeithamova, 2018), with theoretical proposals arguing for an intrinsic link between episodic memory and concept formation (Mack et al., 2018). One reason why the hippocampus was not identified in the experiment by Viganò and Piazza (2020) may be that the new category knowledge had already become consolidated into cortex over the nine days of training, and therefore was no longer hippocampally dependent (Squire et al., 2015). Consistent with this, studies that showed hippocampal involvement in concept learning tested participants relatively early in the learning phase (Davis et al., 2012; Mack et al., 2016). On the other hand, involvement of the EHC in representing conceptual information after long periods of learning as show in Viganò and Piazza's study can inform the debate about the possible role of the MTL structures in organizing semantic knowledge. Future studies could clarify the differential contributions of these MTL structures by tracking neural representations at multiple stages of learning, before and after consolidation.

Another way to uncover the neural representations underlying abstract concepts is to develop computational models that also learn categories (Nosofsky, 1986; Kruschke, 1992; Nosofsky et al., 1994; Ashby et al., 1998; Smith and Minda, 1998; Love et al., 2004; Sanborn et al., 2010), and search the brain for representations that match those learned by the models. This approach has focused on the hippocampus (Mack et al., 2016; Bowman and Zeithamova, 2018) though a recent model based on clustering theories of concept learning was able to capture both hippocampal representations in category learning tasks and place and grid cell-like representations in navigation contexts (Mok and Love, 2019). Applied to a concept structure similar to the one in the current study, the model would learn the centers of the concept distributions (representing the category prototypes), and predict stronger activity for category prototypes than non-prototypes, and more similar representations for movements between category prototypes compared to movements toward non-prototype exemplars. This prediction could not be tested in the current study because the exemplars were equally distant from their category prototype. By expanding the sensory space to include more exemplars and testing more movement directions, future studies may be able to distinguish this model from others, by comparing the similarity spaces within brain regions with similarity spaces implied by different computational models (e.g., spatial, clustering, exemplar, Bayesian models).

While brain-imaging studies provide growing evidence for general-purpose neural mechanisms across spatial and conceptual domains, an older line of behavioral work challenges these ideas by showing that people violate axiomatic assumptions in geometric theories of conceptual organization (Tversky and Gati, 1982). If conceptual spaces are represented geometrically (Gärdenfors, 2000; Bellmund et al., 2018), then they must obey several geometric properties, such as the “triangle inequality.” This states that a direct path between two points cannot be larger than an indirect one going through a third point. Using two-dimensional stimuli similar to ones used in neuroimaging studies (e.g., Constantinescu et al., 2016), Tversky and Gati (1982) showed that people's judgments of the dissimilarity between pairs of exemplars imply internal conceptual spaces that violate the triangle inequality. While augmented geometric models have been developed to address this problem (Krumhansl, 1978; Nosofsky, 1991), some theoretical issues remain (Goldstone and Son, 2012). Therefore, proposals that suggest that spatial representations underlie human concept learning need to address this challenge to resolve the tension between neural data and human behavioral data.

In summary, Viganò and Piazza (2020) provide an important contribution to the growing evidence for shared neural mechanisms underlying spatial navigation and abstract knowledge. Future paradigms should further disentangle the contributions of the hippocampus, the EHC, and the mPFC to the representation of category vs. sensory spaces, assess learning and consolidation-dependent changes in neural representations, and compare computational models in their ability to match neural and behavioral data. Together, these developments will clarify the extent to which we can generalize mechanistic insights from spatial navigation to the organization of higher-order knowledge structures.

## Author Contributions

LB, RH, and RM contributed to the conceptual argument developed in the opinion piece. LB and RM wrote the first draft of the manuscript. All authors contributed to manuscript revision and read and approved the submitted version.

## Conflict of Interest

The authors declare that the research was conducted in the absence of any commercial or financial relationships that could be construed as a potential conflict of interest.
